# Case report of holocarboxylase synthetase deficiency (late-onset) in 2 Chinese patients

**DOI:** 10.1097/MD.0000000000019964

**Published:** 2020-05-01

**Authors:** Zihong Xiong, Guoying Zhang, Xiaoli Luo, Ning Zhang, Jing Zheng

**Affiliations:** Department of Pediatrics Intensive Care Unit, Chengdu Women's and Children's Central Hosptial, School of Medicine, University of Electronic Science and Technology of China, Chengdu, People's Republic of China.

**Keywords:** biotin, holocarboxylase synthetase, holocarboxylase synthetase deficiency, hyperglycemia, mutation

## Abstract

**Rationale:**

Holocarboxylase synthetase (HCLS) deficiency, especially the late-onset type, is a rare disease. Affected patients can present with irreversible metabolic acidosis and may be misdiagnosed with a glucose metabolic disorder. Prompt and correct diagnosis and treatment can reduce mortality to a great extent.

**Patient concerns:**

We report 2 Chinese patients who were diagnosed with late-onset HCLS deficiency. The age of onset of the 2 patients was approximately 8 months. The 2 patients had skin lesions, severe profound metabolic acidosis, dyspnea, and hyperglycemia.

**Diagnoses:**

The results of urinary and blood organic acid analysis with gas chromatography/mass spectrometry revealed multiple carboxylase deficiency. Maple syrup urine disease and diabetic ketoacidosis could not be excluded. This finding is different from those of hypoglycemic complications reported in previous reports. Human genetic analysis eventually provided a definite diagnosis.

**Interventions:**

Prompt oral treatment with biotin dramatically corrected the metabolic imbalances of the 2 patients, and continued oral biotin therapy was essential to the improvement of their prognoses.

**Outcomes:**

Their metabolic disorders were corrected within 48 hours. During long-term follow-up, the patients achieved developmental milestones.

**Lessons:**

Late-onset HCLS deficiency may present with obvious hyperglycemia. Human genetic analysis eventually provided a definite diagnosis. Prompt treatment with biotin is vital to correct metabolic imbalances, and continued therapy is essential to the improving long-term prognoses. Their mutations were p.R508W and c.1088T > A, and these mutations might represent hot-spot genes in Chinese populations with HCLS deficiency. The variants c.1484T > G(p.L495∗) and c.835G > T(p.E279x) are likely pathogenic, and more studies are needed to confirm these results.

## 1 Introduction

Multiple carboxylase deficiency (MCD) is an autosomal recessive disorder of biotin metabolism.^[1]^ The underlying mechanism is biotinidase (BT) or holocarboxylase synthetase (HCLS) deficiency. Deficiency in HCLS results in a decrease in the activity of biotin-dependent carboxylases in the biotin cycle.^[2]^ Clinical manifestations include profound metabolic acidosis, skin rashes, hypotonia, seizures, respiratory distress, hair loss, and developmental delay followed by coma and death if left untreated. The reliable diagnosis of HCLS deficiency requires blood enzymatic determination and genetic analysis.^[3]^

## 2 Case report

### 2.1 Case 1

The first patient was an 8-month-old female infant. She was the second child of healthy unrelated Chinese parents. Her gestation and delivery were uneventful. Her brother was in good health. At the age of 5 months, she presented with erythematous dermatitis localized in the face, neck, and buttock. Over the following months, she was treated with topical corticosteroid and a dairy-free diet for atopic dermatitis with no clinical improvement. Her psychomotor development was slightly delayed, while her nutritional state was normal. From the age of 7 months, she had recurrent respiratory infections. Her condition continued to deteriorate, and she was treated with medicine without improvement. She presented to the ER with an episode of lethargy and shortness of breath, and she was immediately transferred to the pediatric intensive care unit. On admission, an arterial blood gas analysis showed severe metabolic acidosis (pH = 6.98 and base deficit of -25). Biochemical examination found hyperlacticaemia (8.89 mmol/L), hyperammonia (132 μmol/L), and hyperglycemia (15.9 mmol/L). Based on the profound acidosis, metabolic testing was performed (urinary and blood organic acid analysis with gas chromatography/mass spectrometry . Over the next 2 days, the patient's acidosis failed to improve, and respiratory failure and coma ensued. Moreover, the patient developed liver dysfunction, hypoalbuminemia, and sepsis. She was given ventilator support, sodium bicarbonate infusion, and antibiotics. On the fifth day after admission, her urinary organic acid analysis found remarkable elevations in urinary lactate, 3-oxy-butyric acid, 3-OH-isovalerate, 3-OH-propionate, 3-methylcrotonyl glycine, and methylcitrate. In addition, we also found that urinary levels of 2-OH-sovalericacid, 2-keto-isovalericacid, 2-keto-3-methvlvaleric acid, 2-keto-isocaproic, and acetylglycine were higher than normal levels. These findings, combined with elevated levels of leucine and valine in blood, led to a presumptive diagnosis of MCD or maple syrup urine disease The patient was started on oral biotin supplementation (30 mg/d). Six hours later, lactate levels dramatically decreased to 2.7 mmol/L. Over 24 hours, the consciousness of the patient improved rapidly, and the patient was then weaned off ventilation support. On the thirteenth day after admission, the patient was completely off all support, and her skin lesions had improved. The patient was discharged home with continuing biotin treatment. MRI showed changes consistent with HCLS deficiency: mild diffusion limitation changes in the cerebral subcortex, external capsule, and globus pallidus. The patient's condition has been followed regularly, and she must continue taking 10 mg of biotin orally every day. Her mutation was confirmed via medical exome sequencing, which showed she is heterozygous for c.1522C>T(p.R508W). The variant c.1484T > G(p.L495∗) has never been previously reported.

### 2.2 Case 2

The second patient is now a 4-year-old boy who was born at term after an uncomplicated pregnancy and normal vaginal delivery. He was the first child of healthy unrelated Chinese parents. The family history was noncontributory. Motor development was normal, and his weight was lower than that of normal children of the same age. Mild skin manifestations were reported from the first month of life. He presented with erythematous lesions in the face, neck, and buttocks, which were treated with topical steroids without improvement. At 7 months, he was admitted to the pediatric intensive care unit because of deep breath and respiratory distress. An arterial blood gas analysis revealed metabolic acidosis (pH = 7.14 and base deficit of –18). The serum lactate level was high (8.89 mmol/L). He had a transient elevation in random blood sugar (23.68 mmol/L). Blood ammonia levels were normal. The urine was positive for acetone, leading to a suspected diagnosis of diabetic ketoacidosis. After regulation with insulin, the glucose disorder was corrected, but metabolic acidosis continued. The acylcarnitine profile and urinary organic acid analysis showed elevated levels of C-3, C5-OH, propionate, and 3-MCC, respectively, leading to a presumptive diagnosis of MCD. Then, the child was prescribed high doses of oral biotin (30 mg/d). Three days after treatment was initiated, his dyspnea was markedly alleviated, and metabolic acidosis was corrected. Follow-up after 2 months revealed a dramatic improvement in weight gain and skin lesions. Presently, at the age of 4 years, he has remained on biotin (5 mg/d) and has had no delays in development milestones. Genetic analysis revealed that the mutant allele was c.1088T > A(p. V363D). The variants c.1484T > G(p. L495∗) and c.835G > T(p. E279x) are likely pathogenic factors.

## 3 Discussion

Four biotin-dependent carboxylases are found in humans. HCLS catalyzes the process by which biotin is covalently bound via its carboxyl group to an inactive apocarboxylase, forming the active holoenzyme.^[4]^ HCLS deficiency is a rare autosomal recessive disorder of metabolism. HCLS absence or deficiency impairs gluconeogenesis, fatty acid metabolism, and amino acid catabolism and causes dermatological, metabolic, respiratory, and neurological abnormalities. The age of presentation can be variable, and more than half of cases of HCLS deficiency manifest in the newborn period. BT deficiency always appears later. The onset time depends on whether the deficiency is partial or total. HCLS deficiency is more common in China than in other regions,^[5]^ and the dermatologic lesions are more serious and the neurological damage always less severe than that found in BT deficiency. The 2 patients in this study had a relatively late clinical presentation. When they first became symptomatic, they had severe profound metabolic acidosis, dyspnea, and hyperglycemia. An interesting finding from the report of these patients was the obvious glucose metabolic disorder. The first suspected diagnoses in the 2 patients were maple syrup urine disease and diabetic ketoacidosis. Patient 2 had received insulin treatment once. To date, no similar reports have been published. We do not have a satisfactory explanation as to why the 2 patients had profound glucose metabolism disorder, and more studies are needed to evaluate this finding. The diagnosis of HCLS deficiency was confirmed by genetic analysis. Oral biotin was given to both patients at 30 mg/d in the initial stage. Both the clinical and biochemical symptoms were dramatically resolved with oral biotin. The effect of biotin treatment was satisfactory. Their metabolic disorders were corrected within 48 hours. During long-term follow-up, biotin was able to significantly improve the patients’ symptoms and allowed them to achieve normal developmental milestones.

The p.R508W and c.1088T > A mutations have been described in previous reports. These mutations might represent hot-spot genes in Chinese populations with HCLS deficiency.^[6]^ Both mutations are in the HCLS biotin-binding domain.^[7]^ The p.R508W missense mutation has been found in different ethnic groups and belongs to different haplotypes, suggesting a recurrent mutation mechanism.^[8]^ The in vitro expression analysis confirmed that the residual enzyme activity caused by the p.R508W mutation was higher than that of other mutations and had a better response to biotin therapy. At the same biotin concentration, the residual enzyme activity caused by the c.1088T > A mutation was lower than that of the p.R508W mutation, a finding that was also confirmed by in vitro expression analysis, and the c.1088T > A mutation was moderately responsive to biotin therapy.^[9]^ This information will certainly help facilitate the diagnosis and treatment of HLCS deficiency in China.^[10]^ The variants c.1484T > G(p.L495∗) and c.835G > T(p.E279x) are likely pathogenic factors, although additional studies are needed to confirm these findings.

## Author contributions

**Data curation:** Guoying, Jing Zheng.

**Resources:** Zihong Xiong, Guoying Zhang, Xiaoli Luo, Ning Zhang.

**Writing – original draft:** Zihong Xiong.

**Writing – review & editing:** Zihong Xiong, Guoying Zhang.
